# Intelligence

**Published:** 1832-01

**Authors:** 


### INTELLIGENCE.

### THE "BURKING" SYSTEM.

It is with no ordinary regret that we place upon our pages a term which will henceforth be a blot in our language, while the mere mention of it must produce feelings of the deepest abhorrence at the demoniacal atrocities which it brings to the recollection. We are not, however, without strong hopes that the detection of the horrible crimes which have recently been so justly punished will at length compel the legislature of the country to adopt some means that may facilitate the study of anatomy, and at the same time relieve the English professors from the degrading necessity of holding any sort of communion or traffic with those who "men in form, are but brutes by nature." That our expectations are not premature, may be inferred from the promptitude with which Mr. Warburton has brought forward a Bill "for the Regulation of Dissection in Schools of Anatomy." The editors of some of the daily Journals have committed themselves in more than one way upon the subject of the recent murders: it has, in the first place, been asserted that the study of anatomical engravings and wax models might supersede entirely, or at least in a great measure, the practice of dissecting the human body. As we address ourselves to professional readers, we need not formally oppose such absurd opinions. It may be true that he who has once made himself master of the structure of the human frame by dissection, may repair his fading knowledge by either of the plans suggested, but neither can ever suffice to communicate that original anatomical information which *must* be possessed by every practical surgeon and physician.

From the same quarter, too, anatomical professors and surgeons in general have been stigmatised by insinuations, little short, indeed, of positive accusations, that they wink at the commission of murder to ensure an adequate supply of subjects. One Journal, in particular, has long been the Herald of the most ill-natured and unjust sarcasms against us: individual blunders have been stretched into proofs of the general ignorance of the profession, "*notwithstanding the sacrifices which have been made to the interests of science;*" and, in fact, every opportunity has been embraced of prejudicing us in the estimation of the public. It has been urged, and we fear with too much truth in some instances, that a very reprehensible degree of carelessness has existed in the whole system of purchasing bodies for dissection; but let it be remembered that, until the crimes which stain the time in which we live were brought to light, the possibility of their commission was never contemplated; for there are limits within which the most suspicious and cautious minds confine the depravity of human nature. We are confident that in future no surgeon will purchase a body for dissection without the most careful scrutiny, and that, if the *slightest* suspicion arises, the same prompt and decisive steps will be taken, which reflect so much credit upon Mr. Mayo and Mr. Partridge.

We have received the following letter from Mr. Mayo, respecting the murder of the Italian boy:

*To the Editor of the London Medical and Physical Journal.*

Sir: Two accounts have been published relating to the manner in which the supposed Italian boy was destroyed; one as the opinion of the medical witnesses examined upon the trial; the other, as the confession of the murderers. The first appears to have obtained general credit; the second, which nevertheless is singularly and horribly circumstantial in its details, is equally disbelieved. The medical witnesses assert that the death of the boy was occasioned by blows upon his back and neck; the proof of his having received which consisted in the discovery of extravasated and clotted blood in the muscles of the back and neck, and upon the theca vertebralis. The murderers declared that no such violence had been used; that the boy had been destroyed by drowning; and that the appearances above referred to resulted from bending the body after death. In examining this point, upon which the medical witnesses and the murderers are at issue, permit me first to copy a report which it fell to me to lay before the Council of King's College on the 11th of November, six days after the apprehension of the murderers. The first part of this report contains a statement of the appearances which led me, in concurrence with Mr. Partridge, to give into the custody of the police, Bishop, Williams, May, and Shields; the second part contains an account of the internal appearances which I remarked when I made the examination of the body of the Italian boy, in the presence of Mr. Partridge, Mr. North, Mr. Beaman, and Mr. Douchez.

*Report, &c. by* HERBERT MAYO, F.R.S., *Anatomical Professor of King's College.*

The circumstances which led to a suspicion that the boy had been destroyed by resurrection men are the following: The body appeared perfectly fresh, as if it had been living within thirty-six hours; it bore no trace of *lingering* disease; no marks of those remedies which are commonly applied in *acute* diseases; and no signs of any of the kinds of violence which belong to the different forms of *death through accident*: on the other hand, the face was full of blood, the eyes were suffused, the tongue was swollen; one hand, too, appeared slightly swollen and turgid with blood, and the wrist above had a mark, as if it had been compressed. There was no bruise upon the body, or mark of compression about the neck; the forehead had been cut, but the wound had the appearance of having been inflicted after death.*

Upon examining the body, I found no traces of ordinary acute disease in the brain, heart, or bowels. The only deviation from the healthy appearance in the viscera was the following: there were two ounces of turbid water in each pleura, and half an ounce in the pericardium. The substance, likewise, of the lungs was slightly loaded with serous fluid, so that the lungs did not collapse as usual upon opening the chest. It is scarcely possible, however, to suppose that this state of the lungs could have produced death.

There was a considerable quantity of clotted blood in the substance and in the interstices of the muscles of the neck and shoulders; clotted blood was likewise found upon the theca vertebralis in the neck, and in less quantity for a small extent in the scalp at the crown of the head. I believe that these appearances might have been produced by violence done to the body *shortly* after death, as certainly as by violence used during life.

The stomach contained a hearty meal, which, from the degree of digestion it had undergone, had probably been taken from two to three hours before death.

From all these circumstances combined, I am inclined to conclude that the boy's death was sudden, and occurred while he was nearly in perfect health; and that the body must have been, quickly after death, forcibly thrust into a sack or box, with the head downwards, and the spine thus bent and bruised; if, as it is too horrible to suppose, some violence of this kind were not the principal means used to destroy the boy.

HERBERT MAYO.

*King's College; Nov. 10th,* 1831.

I quote this report in order to shew that, *antecedently to the confession of the murderers,* I had concluded, from the examination of the body of their victim, 1st, that the extravasation of blood in the back and neck of the body *was not produced by blows;* and, 2dly, that it was not impossible that this appearance had been produced by force *used after death.* One of several circumstances which led me to the second conclusion was the entire absence of *bruises of the skin* upon the neck or back, such as I believe must have attended the infliction of blows violent enough to have done the mischief found within.

The correctness of the impressions which I had thus formed appears to me to be confirmed by some appearances met with in the body of Bishop, during its dissection in King's College, which were very similar to those in question. The hamstring muscles of the right thigh were at two points partially lacerated, and *the torn texture was loaded with coagulated blood.* In the body of Bishop, as in that of the Italian boy, the blood was perfectly fluid in the vessels, having coagulated only where it had become extravasated.

* We were present at the dissection of the body, and remember that there was some difference of opinion upon this point. Mr. Mayo stated it to be his opinion that the wound had been made after death, and the subsequent confession of Bishop shews this to have been the fact. Two surgeons who were present inferred, from a blush of red which was very evident on the periosteum immediately under the wound, that it was made during life.—EDITOR.

Now, it is evident that the appearance described in the thigh of the murderer cannot be attributed to blows inflicted during life: the injury, we must suppose in this case, was caused either by violence used by the executioner, while death was taking place, or by accidental violence afterwards, such as might have occurred in cutting down the body or removing it. The first of these suppositions appears to me extremely unlikely, considering the prodigious strength which living muscles possess; and I certainly am inclined to adopt the second, and to view the appearances in the body of Bishop as corroborating the opinion that those of a similar nature found in the Italian boy were produced by force used after death.

To what, then, upon this supposition, are we to attribute the death of the Italian boy? It will be seen by reference to my report, that I could form, at the time when I made it, no definite conjecture as to the manner of the murder: now, however, that we have the confession of the murderers, the whole appears sufficiently plain to me. I certainly observed nothing in the body of their victim essentially inconsistent with their story. It is true the heart was small and empty, but the blood was fluid, and the chest was not opened till after I had examined the brain and spinal marrow, the body being laid upon the table with the face downwards. Altogether, I should say that I see no sufficient reason for doubting that the boy was destroyed by drowning, and every reason for disbelieving that he was killed by blows upon the neck.

I remain, Sir, your obedient servant,

HERBERT MAYO.

19, *George street, Hanover square;*
*Dec.* 21, 1834.

*The appearances observed upon examining the body of Bishop were the following:*

The examination took place upon the Wednesday morning, forty-eight hours after the murderer had been executed; during half of that time the body had been in rooms heated by the immense concourse of persons whom curiosity had led to look at it: accordingly some degree of decomposition had begun to shew itself, which was apparent in the dull and opaque cornea, in the state of the viscera of the abdomen, and in the want of consistence in the brain.

The mark of the cord was distinct around the neck, and the skin, when cut through at this part, with the cellular membrane beneath, was found in a state of ecchymosis. The face and scalp were turgid; the eyes bloodshot and prominent; the scalp was greatly loaded with serum.

The vessels of the dura mater and of the cerebrum were not unusually turgid; there was some perfectly thin serum between the arachnoid and pia mater, upon the hemispheres of the brain; and the ventricles were distended with the same secretion. Upon cutting into the substance of the brain, it seemed internally rather more vascular than usual. The surface of the cerebellum and of the pons varolii were suffused with blood; the small vessels upon the surface of these parts being unusually fresh and distinct, and here and there such a general stain of red on the surface as seemed to imply the rupture of vessels.

The heart was large, and contained little blood, and the great veins entering into it were not distended; its chambers were not contracted. The blood was perfectly fluid; no coagulum was met with at any part.

The lungs were perfectly healthy. There was not a drop of fluid in either pleura. There were about two drachms of pale serum in the pericardium. The stomach was small, and contained some chymified mucus. The bowels appeared perfectly healthy.

---

### *The* New Styptic *of* MM. Talrich *and* Halma-Grand.

For some time past we have heard much of the extraordinary influence which a styptic, of unknown composition, possesses in arresting hemorrhages, even from arteries of considerable size: a few days ago, we had the opportunity of personally witnessing its effects. A sheep having been provided, MM. Talrich and Halma-Grand laid bare the carotid artery, and made into it an incision of considerable magnitude, from which the blood was permitted to escape for about a minute. A compress of loose cotton, well soaked in the "Liquide Hemostatique," was then applied to the wound, and retained there for ten minutes by the finger: the hemorrhage was effectually stayed, so that, after that period, the animal was turned loose into the yard, and in fact hunted about, without any recurrence of the hemorrhage whatever.

We cannot, from our own knowledge, determine what would be the effect of this styptic employed in hemorrhages from the human subject; nor do we precisely know what are the effects of *ordinary* styptics when applied to wounded blood-vessels in brutes; but, as the above result is new to us, we communicate what we have seen to our readers.

M. Halma-Grand informs us that his styptic is just as effectual in restraining the flow of blood from wounded veins as wounded arteries; and, as a proof of the confidence he has in its power of arresting hemorrhage in man, he solicits permission to try it after amputation of the thigh or any other limb.

It is stated in the Bulletin Général de Therapeutique, 6me livraison, that the above-named gentlemen have deposited with the French Academy of Sciences a sealed packet, containing the receipt for their "Liquide Hemostatique;" which is not, however, to be opened until they have concluded their experiments in the use of it. From the same Journal we learn also that the facts in favor of its powers are quite conclusive. "Already fifteen sheep have had the carotid artery opened in public; four longitudinally, nine transversely, and in two an oval portion of the artery was removed. In all these instances the hemorrhage was arrested in four or five minutes, and complete cicatrization occurred in a few days. The same result was obtained upon a horse, whose carotid artery was opened at the abatoir of Montfaucon." The editor relates the following case of the successful employment of this styptic in the human subject. A young man had had one of the molar teeth extracted: hemorrhage followed from one of the alveolar arteries of the lower jaw, and, before he was visited by the medical practitioner, he had lost several pounds of blood. Compression and various other means were ineffectually tried. At nine in the morning, a plug soaked in the liquid was placed upon the bleeding point, and another upon the external margin of the lower jaw; they were maintained in these positions with the fingers for some moments, and in seven minutes the bleeding had entirely ceased.

Any of our readers who are curious upon this subject, may obtain from MM. Talrich and Halma-Grand, who are at present at 32, St. James's street, all the information they can require. These gentlemen were kind enough to shew us numerous specimens of portions of wounded arteries from animals, which illustrate the peculiar effects exerted upon bleeding vessels by the application of their styptic. We shall shortly see some other experiments upon this interesting subject, and the result of them will, of course, be communicated to our readers.

---

### LITERARY INTELLIGENCE.

*New Edition of* "DENMAN'S *Introduction to Midwifery*," *by* Mr. WALLER.—At the period when "Denman's Introduction" appeared, it was decidedly the best practical work that had ever been published on the science of obstetrics: the great characteristics of the author were precisely those which command the respect and attention of a reader, a strict adherence to fidelity in the narration of practical facts, and a simple, yet clear and even forcible, style of relating them. The principles of the science of midwifery, as far as they were then known, were also most clearly laid down by Denman. The subsequent improvements in the obstetric department have been, however, numerous and important; and Denman's Introduction, as it was originally published, although perhaps a safe, would not now be a satisfactory guide to the student, or book of reference for the practitioner: to render it adequate to either of these purposes, the valuable practical additions and theories of later writers must be added. This task, we are happy to find, has been undertaken by Mr. WALLER, who is well known to the profession as an eminent and experienced lecturer and practitioner of midwifery. Mr. Waller's connexion with a large midwifery institution, and his extensive private practice in this department of medicine, will doubtless enable him to bring out a work which will prove of much value to the profession, while it is creditable to his own ability.

An Essay on the Nature and Treatment of the Indian Pestilence, commonly called Cholera. By HENRY PENNECK, M.D.—Pp. 40. Highley, London.

Of Pestilential Cholera; its Nature, Prevention, and Curative Treatment. By JAMES COPLAND, M.D., Consulting Physician to Queen Charlotte's Lying-in Hospital, &c.— 8vo. pp. 174. Longman, London.

---

### METEOROLOGICAL JOURNAL,

By Messrs. HARRIS and Co. Mathematical Instrument Makers, 50, High Holborn.

| | Moon. | Rain gauge. | Thermometer. | | | Barometer. | | De Luc's Hygrometer | | Winds. | | Atmospheric Variation. | | |
|---|---|---|---|---|---|---|---|---|---|---|---|---|---|---|
| Nov. | | | 9 a'm. | max. | min. | 9 a.m. | 10 p.m. | 10p.m | 9a.m. | 9 a.m. | 10p.m | 9 a. m. | 2 p.m. | 10 p.m. |
| 20 | | | 47 | 53 | 45 | 29.54 | 29.41 | 66 | 70 | W | W | fine | fine | rain |
| 21 | | | 53 | 56 | 53 | .47 | .64 | 68 | 68 | WNW | WSW | sho'ry | cloudy | cloudy |
| 22 | | .27 | 56 | 57 | 54 | .66 | .80 | 68 | 69 | WSW | W | rain | — | — |
| 23 | | | 56 | 56 | 50 | .78 | .82 | 69 | 68 | WSW | WSW | foggy | — | — |
| 24 | | | 54 | 55 | 48 | .85 | .85 | 68 | 68 | WSW | WSW | fine | fine | fine |
| 25 | | | 52 | 55 | 51 | .76 | .70 | 68 | 69 | SW | WSW | — | cloudy | — |
| 26 | ☾ | | 52 | 56 | 45 | .80 | .95 | 68 | 66 | N | E | cloudy | — | cloudy |
| 27 | | | 49 | 53 | 35 | 30.16 | 30.24 | 63 | 63 | NE | NE | fine | fine | fine |
| 28 | | | 38 | 40 | 37 | .29 | .31 | 61 | 60 | W | N | foggy | — | cloudy |
| 29 | | | 40 | 40 | 36 | .35 | .33 | 60 | 62 | W | SW | — | foggy | foggy |
| 30 | | | 40 | 45 | 40 | .12 | 29.98 | 62 | 66 | W | NW | — | rain | cloudy |
| Dec. | | | | | | | | | | | | | | |
| 1 | | .10 | 45 | 48 | 45 | 29.98 | .94 | 67 | 67 | NE | NNW | — | cloudy | — |
| 2 | | | 48 | 48 | 42 | .85 | .89 | 67 | 66 | N | NW | fine | fine | — |
| 3 | | | 45 | 49 | 38 | .98 | .97 | 66 | 65 | NW | W | foggy | cloudy | — |
| 4 | ● | | 42 | 46 | 45 | .68 | .68 | 69 | 68 | SW | SW | cloudy | sho'ry | rain |
| 5 | | | 44 | 47 | 45 | .63 | .47 | 67 | 67 | WSW | S | — | cloudy | fine |
| 6 | | .35 | 50 | 53 | 50 | .28 | .21 | 67 | 69 | S | S | — | — | cloudy |
| 7 | | | 52 | 55 | 48 | 28.78 | 28.98 | 67 | 68 | SSW | SW | rain | sho'ry | rain |
| 8 | | .45 | 53 | 55 | 52 | .96 | 29.07 | 68 | 69 | SW | SW | — | sleet | — |
| 9 | | | 54 | 55 | 48 | 29.02 | .13 | 69 | 69 | SW | WSW | cloudy | cloudy | fine |
| 10 | | .40 | 52 | 53 | 51 | .24 | .45 | 69 | 68 | SW | SSW | — | fine | — |
| 11 | | | 54 | 56 | 49 | .53 | .32 | 71 | 71 | SW | SW | — | sho'ry | cloudy |
| 12 | ☽ | | 52 | 55 | 50 | .12 | .05 | 68 | 65 | S | SW *v.* | rain | rain | rain |
| 13 | | .34 | 53 | 54 | 46 | .25 | .39 | 63 | 66 | SW | SW | fine | fine | fine |
| 14 | | .08 | 49 | 50 | 40 | .41 | .47 | 67 | 66 | SW | W | — | — | — |
| 15 | | | 41 | 44 | 40 | .62 | .52 | 66 | 65 | W | SW | — | — | — |
| 16 | | | 46 | 50 | 42 | .66 | .26 | 65 | 67 | SW | SW *v.* | foggy | — | rain |
| 17 | | | 45 | 49 | 44 | .58 | .57 | 65 | 65 | WSW | SSW | — | — | — |
| 18 | | | 45 | 47 | 41 | .43 | .25 | 67 | 66 | SW | SW | cloudy | cloudy | fine |
| 19 | ☉ | .56 | 42 | 44 | 41 | .41 | .55 | 64 | 65 | WSW | W | foggy | fine | — |

The quantity of Rain fallen, in the month of November, was 93.100ths of an inch.

